# Artificial Intelligence in Scientific Writing: A Deuteragonistic Role?

**DOI:** 10.7759/cureus.45513

**Published:** 2023-09-18

**Authors:** Benny Wohlfarth, Samuel R Streit, Sissel Guttormsen

**Affiliations:** 1 Institute for Medical Education, University of Bern, Bern, CHE; 2 Department of Angiology, Inselspital Bern, Bern University Hospital, Bern, CHE; 3 Institute of Medical Education, University of Bern, Bern, CHE

**Keywords:** ai regulation, large language models (llms), chatbots, swa (scientific writing assistance), research integrity

## Abstract

In this article, we reflect on the pros and cons of artificial intelligence (AI)-augmented scientific writing for more comprehensible research towards society to gain trust for science-led policy. For this purpose, we integrated our thoughts into the *Factors of Perceived Trustworthiness from Mayer, Davis, and Schoorman’s Model of Trust* and made propositions to define AI’s role in trustful scholarly communication.

## Editorial

Letter to the editor

In ancient Greek drama, the deuteragonist is the second actor in the play. While interacting closely with the protagonist to support and assist, the deuteragonist can also assume a role of conflict or rivalry; ultimately helping to develop the main character and shape the overarching narrative of the play [1].

Since artificial intelligence (AI) came to the stage in 1956 [2], it has gained momentum from the 1990s onwards. Today, it affects nearly every aspect of how we live and work as a society, whether in healthcare [3], education [4], economics [5], arts [6], or science [7]. Generally, people have high trust in science [8]. However, this trust is being tested due to incidents of human bias, error, and fraud in production [9-11], interpretation [11,12], and reporting and publication of scientific work [12,13]. Particularly since the COVID-19 pandemic, conflicting scientific results have done little to cultivate greater trust in science [14,15]. For society's acceptance and trust in science to grow, it is essential to make research as transparent and comprehensible as possible to everyone in the "theatron," while maintaining robustness and objectivity [16].

Balancing the benefits and risks of AI in scholarly communication

**Figure 1:**
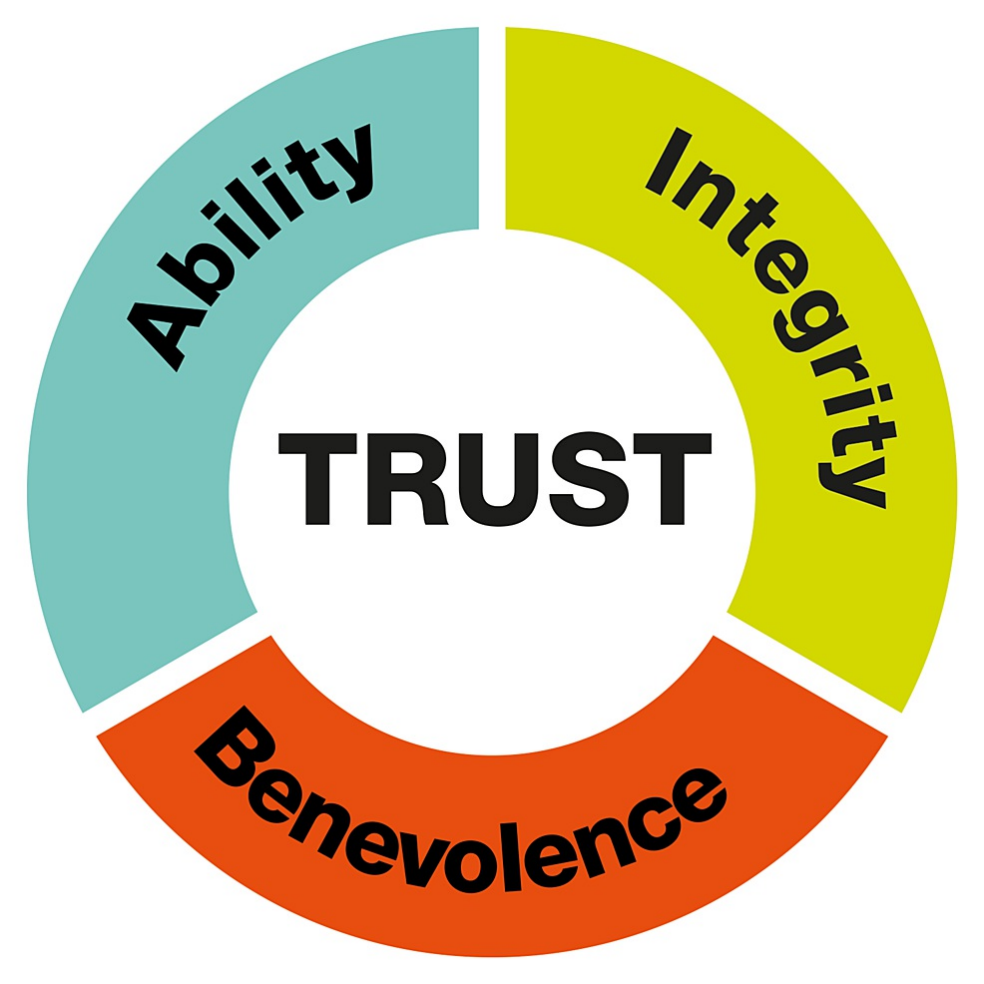
Factors of Perceived Trustworthiness from Mayer, Davis, and Schoorman's Model of Trust *Ability* refers to skills, competencies, and characteristics that enable an individual to be influential in a particular domain. *Benevolence* holds the idea that the trustee has a genuine intention to do good for the trustor, independent of self-centered motives. *Integrity* is the trustor's perception that the trustee adheres to a set of principles deemed acceptable by the trustor [19].

The concept of AI assisting researchers in scientific writing is appealing. If executed correctly, AI could support more comprehensible research and increase acceptance of science-led policy by the general public. However, concerns have been raised about issues such as plagiarism and obscurity in research, particularly with unregulated use of AI-driven scientific writing assistance (SWA) and chatbots [17,18]. In this article, we reflect on the pros and cons of AI in scientific writing. To do so, we integrate these considerations into the Factors of Perceived Trustworthiness from Mayer, Davis, and Schoorman's Model of Trust (see Figure 1) [19]. Originating from the social sciences, this model has proven valuable and robust across various disciplines and is also widely utilized in information systems-related science [20,21]. This background renders it perfect to transfer our thoughts on AI’s role in scientific writing.

The pros

SWA has the potential to make research more accessible and understandable by facilitating core findings for wider target groups, including non-scientific stakeholders, policymakers, and the public (benevolence+) [22]. In addition, SWA and chatbots hold a chance to offer more complex language assistance to non-native English speakers, who may struggle with the costs of language editing services (benevolence+) [23]. This augmentation further improves scientific writing by suggesting simple yet correct sentence structures, enhancing clarity and the overall readability of a manuscript (ability+).

AI can generate various visual representations of research, ranging from simple semiotic visualizations to more complex schematic views. These offer effective and easily comprehensible data communication for other researchers and a broader audience in a non-textual form (ability+).

Humans are prone to errors, especially when performing repetitive and monotonous tasks like formatting citations and proofreading [24]. AI automation for such tasks counteracts decision fatigue and frees time for scientists to focus on the research content, specifically, interpreting and concluding from data, thereby ultimately accelerating scientific progress for society (ability+) [25].

AI can quickly analyze and summarize vast amounts of scientific literature or content data, enabling researchers to uncover patterns, correlations, and trends not easily identifiable through manual analysis alone (ability+). This can also contribute to improve the overall quality of the research process; for example, in identifying relevant research questions and interpreting data, resulting in a more targeted design of original research and literature reviews (benevolence+) [26,27].

The cons and possible measures

Since AI chatbots are based on large language models (LLM), their answers are always a function or a derivate of their repertoire and hold a risk of bias to scientific writing, based on possible biases in the training datasets themselves. This may include racial, sexual, or religious bias, making the results less inclusive [28-30] (integrity^-^). This should be addressed by fostering the establishment of balanced training of datasets, bias detection, and correction algorithms, as well as close human supervision and feedback mechanisms [29,31,32].

Automation of tasks, e.g., the aforementioned SWA, may stoke fear of job loss [33] (benevolence^-^). Mitigating such concerns must be enforced by public dialogue, addressing integrative scenarios including supervising, and collaborating definitions as well as adequate education and reskilling programs for the people in an increasingly AI-automated world.

The crystalline nature of LLMs precludes chatbots from actuality. More importantly, while they are neither living nor experiencing culture, LLM chatbots are put into an insurmountable epistemic distance toward the user’s world of thought, which ultimately makes current chatbots incapable of generating a priori ideas or conclusions for society (integrity^-^, ability^-^). This could be closed by the aforementioned human-AI collaboration, as well as pairing LLMs with augmented reality, evolutionary algorithms, generative models, fine-tuning, and zero-/few-shot learning to generate inclusive novel ideas and writings [34-36].

Overreliance on AI for writing tasks can lead to a decline in critical thinking and writing skills among researchers, as well as an impoverishment of creativity and inclusivity. This could potentially jeopardize the integrity of scientific research (integrity^-^), lowering people’s trust in it, and open the door for predatory journals to misuse AI to mass-produce low-quality, inaccurate, and misleading content, based on monetary interests [37] (benevolence^-^). We suggest the encouragement of education and training in young researchers on critical thinking and writing skills, highlighting that AI is a tool of assistance, not a replacement for the human intellect [37,38] and applaud the viewpoint to see AI more in the sense of an “augmented intelligence” rather than an “artificial intelligence” by successfully partnering with machine intelligence [39]. Furthermore, rules and standards for the use of AI in scientific writing must be agreed on to maintain research integrity and discourage misuse, with possible penalties for infractions [37,38]. The development of advanced AI auditing systems to detect and prevent the mass production of low-quality, inaccurate, or misleading content is needed. Furthermore, strict peer review processes must be a standard, as well as automated checks against known databases. The latter is already possible through various tools that require mandatory human supervision [23,40].

Lack of explainability [41] and the generation of misleading or hypothetical claims and references, known as hallucination [42] are critical topics in regard to society's trust. Unverified adoption of such texts and references can result in legal consequences and ultimately produce false results and conclusions that impede society’s trust in research (integrity^-^). We advise fact-checking and the use of verification systems to confirm the accuracy of AI-generated text before publication or use, predominantly by human reviewers, but also other AI-based systems, as well as the development of AI algorithms specifically designed to detect and correct hallucinations and other errors in AI-generated text [43].

Implementation and effective utilization of AI in scientific writing requires technical expertise and resources. Not all researchers may have access to or be comfortable with using it, potentially creating a split in the scientific community (integrity^-^). Additionally, access to AI tools may be limited due to factors such as cost or institutional constraints (ability^-^, integrity^-^). This could be counteracted by faculties by providing comprehensive training to researchers on the use of AI tools, ensuring that they are comfortable and proficient in their application.

In general, governmental and international organizations should foster the development and promotion of open-source AI models like BLOOM, which holds the opportunity to make AI-based research more inclusive by reducing potential splits caused by financial or institutional constraints.

Future plays: regulation, not restriction

Famous misjudges in the past show that new technology is often exposed to cognitive bias, such as status quo bias [44]. Even experts and opinion leaders plunged into false predictions and disputed the advantages of new technology throughout history [45]. In this sense, the outright rejection of AI in scientific writing is not a solution. Undoubtedly, AI is here and is gaining terrain quickly. OpenAI’s ChatGPT, which was launched in November 2022, exemplifies this technology and its rapid development. With more than 100 million users in the first two months of its release, it is the fastest-growing application in the history of web applications [46].

Ethical Considerations

To define AI's role in the future of scientific writing, our reflection on the pros and cons, combined with suggestions for reducing risks, is an important contribution to this debate. Considering that AI presumably will become more complex and involved in the future, e.g., in the concept of artificial general intelligence (AGI) [34,35], it is crucial to discuss and find consensus on the raised concerns in a timely manner. We see the potential for AI in scientific writing, given that robust and ethical frameworks for the incorporation into the daily work of scientists can be established. After all, it ­­­must preserve the commitment for scientific integrity and trustworthiness towards society and at the same time acknowledge the thirst for innovation.

Limitations of AI for Scientific Writing

The inherent risk of introducing bias, the phenomenon of "hallucination," a lack of originality, limited accessibility, and insufficient transparency and explainability hinder AI from becoming a protagonist in scientific writing at its current technological stage and without clear guidelines.

**Figure 2:**
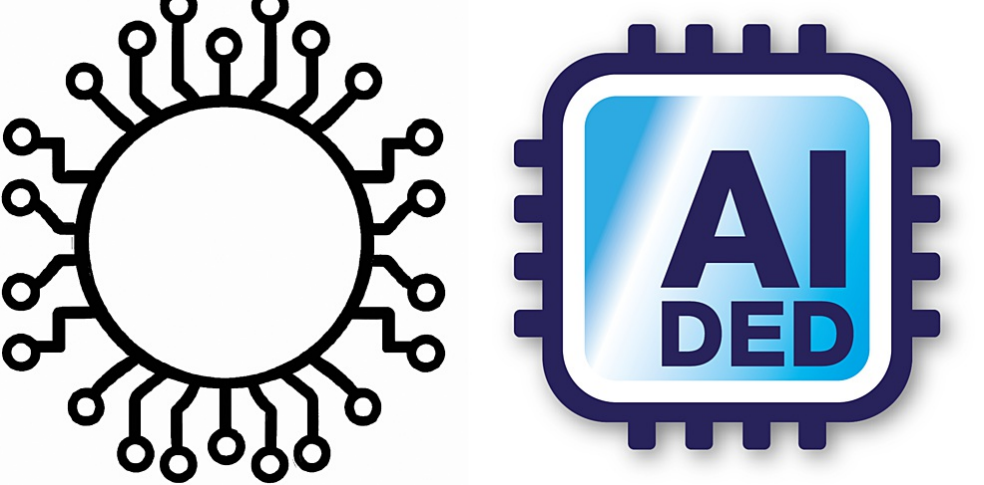
Proposition of badges for labeling AI usage in scientific writing. Left: AI-generated badge by DALL·E on the prompt: “Please make a symbolic logo that could be used as a badge to label text passages in scientific text, that have been generated by AI”, not quite meeting cultural expectations, as the circuits could be negatively associated, for example, with the resemblance of Medusa’s hair. Right: AIDED badge, human creation done in Adobe Illustrator (Adobe Inc., San Jose, California, United States) by the authors.

For now, we advocate the general disclosure of AI usage in the methods section of research articles as an initial step. The declaration should include the type of AI used and clarify the role the AI had in the creation of the manuscript. In addition to that, the corresponding scripts and prompts, as well as chatbot parameters, should accompany the article as supplementals and data repositories. To guarantee full transparency, the following measures should be developed and established: (a) standardized reporting frameworks customized to the AI model in use. This ensures that essential AI-related details are reported consistently and thoroughly, providing additional clarity to the reader. (b) Introduce a visual cue on the published article, in analogy to open access labeling, by creating an international AI usage badge (Figure 2); a visual indicator displayed on studies employing AI, signifying adherence to AI good practices. Akin to the open-access badges, this would allow immediate recognition of AI's use and its compliance with standard transparency guidelines. (c) Scientific databases should have a search option to include or exclude papers with AI involvement in the making of the manuscript. (d) We suggest the introduction of an "AI Statement" section. This would include a comprehensive report detailing why AI was used, its assigned tasks, possible biases of the model, mitigation strategies, and how ethical considerations were addressed. In the sense of multidisciplinarity and in analogy to statisticians, being involved in large and sophisticated analyses, this may include an AI responsible person with an appropriate technical background when AI is heavily involved.

Conclusions

The boundary that separates AI as a mere tool and a potential autonomous contributor - a protagonist, akin to a coauthor - gradually becomes less distinct as its technology and usage evolve. However, the future role of AI as a protagonist in research has to be postponed. For now, AI will remain in the second line, making us protagonists better authors through its help and our consecutive reflection on the rules of good scientific writing for a more comprehensible and trustful scholarly communication.
